# Impact of the Immune Landscape in Follicular Lymphoma: Insights into Histological Transformation in the Rituximab Era

**DOI:** 10.3390/cancers16203553

**Published:** 2024-10-21

**Authors:** Marie Hairing Enemark, Maja Lund Jensen, Maja Dam Andersen, Trine Lindhardt Plesner, Stephen Hamilton-Dutoit, Maja Ludvigsen

**Affiliations:** 1 Department of Hematology, Aarhus University Hospital, 8200 Aarhus, Denmark; mariem@rm.dk (M.H.E.); maja.jensen97@gmail.com (M.L.J.); maja.aner@auh.rm.dk (M.D.A.); 2 Department of Clinical Medicine, Aarhus University, 8000 Aarhus, Denmark; 3 Department of Pathology, Copenhagen University Hospital, 2100 Copenhagen, Denmark; trine.lindhardt.plesner@regionh.dk; 4 Department of Pathology, Aarhus University Hospital, 8200 Aarhus, Denmark; stephami@rm.dk

**Keywords:** follicular lymphoma, histological transformation, gene expression, macrophages, neutrophils

## Abstract

**Background:** Follicular lymphoma (FL) presents significant clinical heterogeneity, with some patients experiencing transformation into an aggressive disease, a key contributor to FL-related mortality. Based on gene expression profiles, this study aimed to provide insights into immunological differences associated with transformation. **Methods:** Gene expression analysis using the NanoString nCounter Tumor Signaling 360 Panel was performed on diagnostic lymphoma samples from 70 FL patients diagnosed in the rituximab era, either non-transforming FL (nt-FL, n = 34) or subsequently transforming FL (st-FL, n = 36), with paired high-grade transformed FL (tFL, n = 36) samples available. In silico immunophenotyping was performed to infer immune cell infiltration using the CIBERSORTx algorithm. **Results:** The gene expression analysis revealed 164 significantly differentially expressed genes, distinguishing st-FL from nt-FL and generally presenting an upregulation of B cell-related genes (CD40, IRF4, RELB), immunosuppressive molecules (IL10, SOCS3), and immune checkpoint molecules (CD276, TIM3). Analysis of immune cell proportions indicated significant differences in infiltrates of M1-like macrophages (*p* = 0.007) and neutrophils (*p* = 0.012) in nt-FL versus st-FL samples. Transformation-free survival (TFS) was associated with high numbers of both these cellular subsets (*p* = 0.006 and 0 = 0.002, respectively). This was even more evident when combined with inferior TFS in lymphomas with high infiltrates of both cell types (*p* < 0.001). After transformation, tFL samples showed a reduction in T follicular helper cells (*p* = 0.008) and an increase in immunosuppressive M2-like macrophages and neutrophils (*p* < 0.001 and *p* = 0.028, respectively). **Conclusion:** By elucidating the distinct molecular and immune landscapes of FL at the time of diagnosis and transformation, this study underscores the importance of immune microenvironment in FL transformation and patient outcome.

## Simple Summary

This study examines the immune differences in follicular lymphoma (FL) and particularly focuses on the transformation into an aggressive disease, a major cause of FL-related deaths. We analyzed gene expression in diagnostic samples from 70 FL patients, categorized as either non-transforming FL (nt-FL) and subsequently transforming FL (st-FL) using NanoString technology. We identified 164 significantly differentially expressed genes between nt-FL and nt-FL samples, with st-FL showing an increased expression of B cell-related and immunosuppressive genes. Particularly, immune cell analysis revealed differences in macrophage and neutrophil fractions between nt-FL and st-FL, with higher levels of these cells linked to shorter transformation-free survival. Meanwhile, tFL samples had fewer T follicular helper cells and more immunosuppressive macrophages and neutrophils. The findings highlight the critical role of the immune microenvironment in FL transformation and patient outcomes.

## 1. Introduction

Follicular lymphoma (FL) is a low-grade lymphoma entity with favorable outcomes despite it being incurable. For most patients, the disease is of indolent character with a slow progression rate and good responses to therapy [1,2]. The overall survival has improved greatly after the introduction of rituximab as standard treatment, yet most patients will experience multiple relapses [1,3,4,5]. Furthermore, some patients will experience histological transformation to an aggressive type, typically with histological features of diffuse large B cell lymphoma (DLBCL). Transformation occurs at a rate of approximately 2–3% per year, which is associated with remarkably poorer clinical behavior and is a key contributor to FL-related mortality [1,6,7,8]. Thus, patients await a future of recurrent relapses, progressive treatment refractoriness, and frequent hospital visits. Accordingly, the clinical spectrum is highly variable due to the heterogeneous presentation of the disease, leading to great variability in outcome [1,4,5].

At present, neither clinical nor molecular markers have been able to assess the risk of transformation at FL diagnosis. Moreover, biology has yet to elucidate causative mechanisms, leading to transformation in this rather great subset of patients [9,10]. Prospective risk stratification of transformation risk would enable patients with low-risk disease to experience an improved general wellbeing of life, while high-risk patients would benefit from more targeted and effective therapies upfront.

The tumor microenvironment (TME) constitutes an essential component in FL, and it has become increasingly evident that the elements of the TME are much more than just bystanders but rather a part of an inflammatory response that plays a crucial role in the onset, progression, and outcome of FL [1,6,11]. Although the tumor-infiltrating immune cells are not of a malignant phenotype, their presence in the TME plays a pivotal role in tumor biology. The importance of components of the TME was demonstrated in a landmark microarray gene expression study by Dave et al., in which specific gene expression and immune signatures from infiltrating immune cells were found to determine patient survival [12]. While several studies have focused on gene expression analyses, only a few gene expression analyses have been studied with transformation as the endpoint, with even fewer investigating samples from patients treated with rituximab [13,14,15,16,17].

Given the relevance of the tumor microenvironment (TME) for malignant cell survival, it is crucial to identify new immune biomarkers associated with disease outcomes in FL. In this study, we aimed to decipher the intricate tumor biology in FL in a national cohort of FL patients diagnosed in the rituximab era by identifying putative differentially expressed genes (DEGs) important to the transformation of FL.

## 2. Materials and Methods

### 2.1. Patients

Analyses were performed on pre-therapeutic formalin-fixed, paraffin-embedded (FFPE) lymphoma biopsies from 70 patients diagnosed with FL grade 1–3A at lymphoma-treating centers throughout Denmark in the rituximab era, between 2005 and 2020. These were either (i) non-transforming FL (nt-FL, n = 34) patients without transformation with at least ten years follow-up or (ii) subsequently transforming FL (st-FL, n = 36) with subsequent histologically confirmed transformation to DLBCL or FL grade 3B at least six months after the primary FL diagnosis to avoid misclassification of discordant/composite lymphoma. For the second group, paired high-grade transformed FL (tFL, n = 36) samples from the time of transformation were also analyzed. All biopsies were reviewed by an experienced hematopathologist and classified according to the 2017 update to the WHO classification [2]. Clinicopathological data were collected from The Danish Lymphoma Registry [18,19].

This study was approved by the Danish National Committee on Health Research Ethics (1-10-72-276-13) and the Danish Data Protection Agency (1-16-02-407-13) and was conducted in accordance with the Declaration of Helsinki.

### 2.2. Gene Expression Analysis

RNA was extracted from FFPE lymphoma specimens using the High Pure FFPET RNA Isolation Kit (Roche, Basel, Switzerland). Two 10 µm FFPE sections were processed according to the manufacturer’s protocol. The concentration of RNA was quantified using Implen NanoPhotometer (N50-Touch, Implen GmbH, München, Germany) according to the manufacturer’s instructions. A total of 100 ng of RNA was required for analysis. mRNA expression was profiled using the NanoString nCounter Tumor Signaling 360 Panel (XT Hs Tumor Signaling 360 CSO, NanoString Technologies, Seattle, WA, USA) according to the manufacturer’s recommendations. RNA was hybridized overnight at 65 °C, and purification and binding of hybridized probes to the cartridge were performed on the nCounter Prep Station (NanoString Technologies). Cartridges were scanned on the nCounter Digital Analyzer (NanoString Technologies). 

The data were analyzed with nSolver 4.0 software (NanoString Technologies, Seattle, WA, USA). The quality of the data was assessed using the default quality control settings, and normalization was performed using the geNorm algorithm within nSolver [20]. Log2-transformed data were used in subsequent data analysis. 

Genes were excluded if they were not above a background count threshold of 20 and present in >50% of samples. Thus, the final analysis included 647 genes in the analyses of nt-FL versus st-FL samples, as well as 659 genes in the analyses of st-FL versus tFL samples. Subsequent data analysis was performed in nSolver (NanoString Technologies) and custom R scripts (RStudio, version 4.4.0).

### 2.3. CIBERSORTx in Silico Immunophenotyping

To infer the proportions of infiltrating immune cells in the lymphoma samples, in silico immunophenotyping was performed using the CIBERSORTx algorithm. This algorithm uses a set of reference gene expression values from bulk tissue gene expression for the profiling of 22 immune cell types (the LM22 signature matrix based on 547 genes) [21,22,23]. Gene expression data were uploaded to the CIBERSORTx web portal (https://cibersortx.stanford.edu/, assessed on 31 August 2024) at 1000 permutations with enabled bulk-mode batch correction.

### 2.4. Gene Enrichment Analysis

Gene enrichment analyses were performed using both the STRING database (string-db.org) using the StringApp (version 1.7.0) in Cytoscape (version 3.10.2) [24,25,26]. For each gene, the corresponding UniProt IDs were submitted to the software tool based on the set of significant DEGs. In both the nt-FL versus st-FL analysis and the st-FL versus tFL analysis, one UniProt ID was not recognized in the Cytoscape STRING software, resulting in a total of 163 and 313 genes for analysis, respectively. The minimum required interaction score was set to medium confidence, 0.4, with both functional and physical interactions included. Enriched terms were filtered to include only terms from UniProt Keywords, Gene Ontology (GO) Biological Processes, GO cellular components, GO molecular functions, Kyoto Encyclopedia of Genes and Genomes (KEGG) Pathways, Reactome Pathways, and STRING clusters [27]. The false discovery rate was set to 5%. The option to remove redundant terms was enabled, and the redundancy cut-off was set to 0.50.

### 2.5. Statistical Analysis

Differences in clinicopathological data were assessed using a chi-squared test or Fisher’s exact test as appropriate. Differences in gene expression were analyzed using a two-tailed t-test. Being explorative in its study design, no further correction was performed in order to not increase type II error and thus risk missing out on putative important markers. Principal component analyses (PCAs) were performed using DEGs with no missing values to avoid imputation. Hierarchal clustering was performed with Euclidean distance as the dissimilarity measure and Ward’s linkage to join clusters. Differences in immune cell proportions between nt-FL, st-FL, and tFL samples were assessed using an independent Mann–Whitney U test and a paired Wilcoxon ranked-sum test. Patient outcomes were analyzed using the Kaplan–Meier and log-rank method with overall survival (OS) or transformation-free survival (TFS) as the endpoint. OS was defined as the time from diagnosis to death from any cause or censoring, while TFS was defined as the time from diagnosis to transformation [27]. *P*-values below 0.05 were considered statistically significant. Statistical analyses were performed using R statistical software (version 4.1.2).

## 3. Results

### 3.1. Patient Characteristics

The patient cohort comprised a total of 70 FLs, and all were diagnosed within the rituximab era, of which 36 st-FL patients experienced subsequent transformation while 34 nt-FL patients did not, as shown in Table 1. For the st-FL patients, in addition to the diagnostic FL sample, a paired high-grade biopsy from the time of transformation was also included. Ages ranged from 35 to 80 years, with a median age of 58 years at diagnosis. Slightly more men than women were included in this study, which was especially evident as the st-FL group encompassed significantly more males than the nt-FL group. No significant difference was found in other of the common clinicopathological features analyzed. 

### 3.2. Immune-Related Genes at Time of FL Diagnosis Associated with Transformation

**Figure 1:**
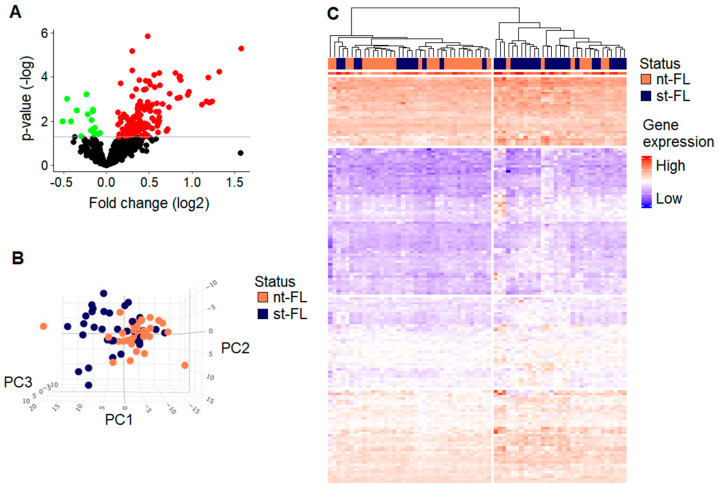
Differentially expressed genes at diagnosis in FL with and without subsequent transformation. (**A**) Differentially expressed genes between diagnostic nt-FL and st-FL samples. Red, upregulated; green, downregulated. The horizontal line marks *p*-values of *p* < 0.05. (**B**) A 3D PCA plot with input of differentially expressed proteins at *p* < 0.05 comparing nt-FL and st-FL samples. (**C**) Hierarchal clustering analysis based on differentially expressed proteins at *p* < 0.05 comparing nt-FL and st-FL samples. Abbreviations: nt-FL, non-transforming samples; PC, principal component; st-FL, subsequently transforming samples.

Gene expression analyses of diagnostic nt-FL and st-FL samples included a total of 647 genes from the NanoString nCounter Tumor Signaling panel, housing a broad panel of genes involved in tumor signaling and immune infiltration within the TME, as shown in Table S1. From these, 164 genes were significantly differentially expressed between the two groups (fold changes of 0.69–2.99). Depiction of the data in a volcano plot showed a general trend of upregulation of the genes in st-FL samples. Accordingly, 148 differentially expression genes (DEGs) were upregulated in contrast to only 16 DEGs that were downregulated in st-FL samples, as shown in Figure 1A.

Differential expression was observed with upregulation of B cell-related genes (*CD40, IRF4* [MUM1], *RELB* [NFκB]) as well as, surprisingly, borderline downregulation of *MS4A1* [CD20]. Samples from patients with subsequent transformation were further implicated by the upregulation of immunosuppressive molecules (*IL10, SOCS3*) and immune checkpoint molecules (*CD276* [B7-H3], *HAVCR2* [TIM3]) as well as borderline upregulation of checkpoints (*LAG3, PDCD1* [PD-1], *PDCD1LG2* [PD-L2]). This was further underlined by the downregulation of genes associated with T helper function (*CD40LG*). Additionally, several of the upregulated genes were associated with cytotoxicity (*CXCL10, FCGR3A/B)* as well as macrophages (*CD14, CD68, CSF1R, IL6, SIRPA*). Moreover, differentially regulated genes involved cell cycle (*CCNB2/E1/F/K, CDC25A, CDK4, CDK6, CDKN1A, CHEK1, FOXM1*), P53 degradation (*MDM2, COP1, AURKA*), cellular migration (*ACTG2, ICAM1, ITGA1/A5/B1, SERPINE1*), and apoptosis (*BCL2A1, CASP3*).

To investigate whether DEGs could differentiate FL samples according to subsequent transformation, unsupervised PCAs and hierarchal clustering analyses were performed. With the input of the significant DEGs, the analysis revealed a strong focus on nt-FL samples alongside a more widespread presentation of st-FL samples, as shown in Figure 1B,C. Notably, especially evident in the hierarchical clustering analyses were two main groups that were observed corresponding to the majority of nt-FL and st-FL samples, respectively, reflecting possible risk subgroups of FL, as shown in Figure 1C.

To describe the most significant differences between the patient groups, a significance threshold of *p* < 0.01 was also applied, resulting in 81 differentially expressed genes. However, this set of genes did not outperform the initial distinction when analyzed by PCA and hierarchical clustering, as shown in Supplementary Figure S1A,B.

### 3.3. Differentially Regulated Genes Reveal Disturbed Biological Pathways Depending on Subsequent Transformation Status

**Figure 2:**
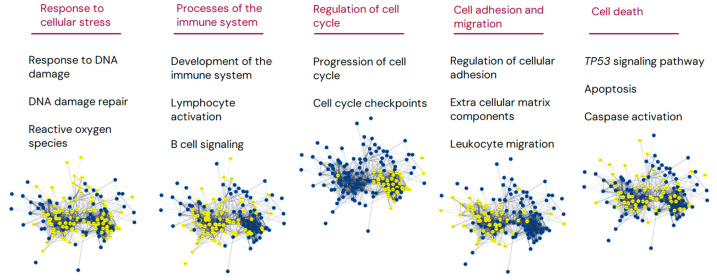
Gene enrichment analysis of significantly differentially expressed genes comparing nt-FL and st-FL samples. Gene enrichment analysis of the set of 164 DEGs comparing nt-FL and st-FL samples. Nodes represent proteins and edges visualize interactions. The depicted groups belong to responses to cellular stress, the immune system, cell cycle signaling, cellular adhesion and migration, and cell death. Yellow indicates proteins involved in the said pathway; blue, proteins are not involved in the pathway.

Gene enrichment analysis was based on the 164 significant DEGs and identified numerous possibly disturbed cellular pathways comparing lymphoma biopsies from nt-FL and st-FL patients, as shown in Figure 2 and Table S2. The enrichment analysis recognized 163 nodes and 1709 edges, leaving a network of significantly more interactions than expected by random (*p* < 0.001).

Interestingly, despite rather subtle changes in gene expression levels (most changes less than two-fold), the pathway analyses revealed possible biological differences in important cellular pathways, including cellular response to stress, processes in the immune system, the regulation of cell cycle, cell adhesion, and migration, and the regulation of cell death. Ultimately, these constitute important pathways may ultimately influence cell growth and survival as well as the non-malignant TME.

### 3.4. Genes Associated with Macrophage and T Cell Infiltration Are Differentially Expressed in Transformed FL

**Figure 3:**
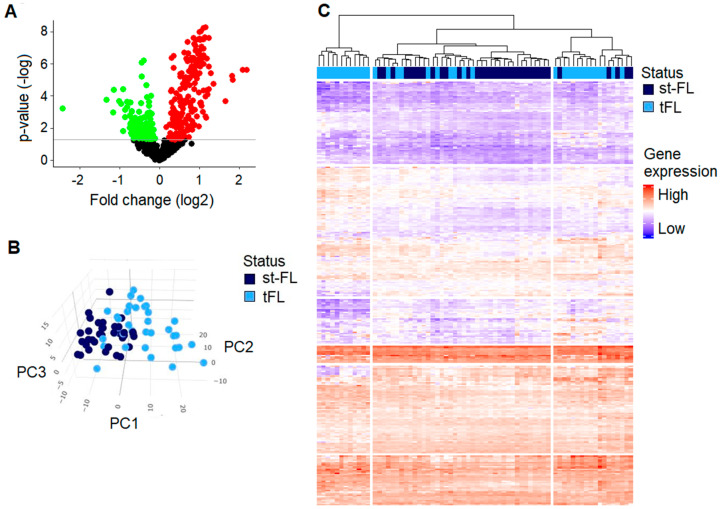
Differentially expressed genes between diagnostic st-FL samples and high-grade tFL samples. (**A**) Differentially expressed genes between diagnostic st-FL and tFL samples. Red, upregulated; green, downregulated. Horizontal line marks *p*-values of *p* < 0.05. (**B**) A 3D PCA plot with the input of differentially expressed proteins at *p* < 0.05 comparing st-FL and tFL samples. (**C**) Hierarchal clustering analysis based on differentially expressed proteins at *p* < 0.05 comparing nt-FL and tFL samples. Abbreviations: PC, principal component; st-FL, subsequently transforming samples; tFL, transformed FL.

A total of 659 genes from the Tumor Signaling panel were included in the analyses between paired diagnostic st-FL samples and high-grade tFL samples. From these, 314 genes were significantly differentially expressed (fold changes 0.19–4.53) and were distributed across 191 upregulated and 123 downregulated genes in the tFL samples, as shown in Figure 3A and Table S3.

DEGs included the particularly indicated downregulation of T cell-related genes (*CCR7, CD3E/D/G, CD247* [CD3 zeta], *CD6, CD28, CD40LG, FOXP3, ICOS, IL7, IL7R, ITK, PDCD1* [PD-1], *TCF7, TGFB2, ZAP70*). This was accompanied by the upregulation of genes related to macrophages, particularly of the immunosuppressive M2-like subtype (*CD14, CD68, CD163, CFS1R, SIRPA*), immune checkpoint molecules (*HAVCR2* [TIM3], *PDCD1LG2* [PD-L2]), and trending associations to even more immune checkpoints (*CD274* [PD-L1], *CD276* [B7-H3]). In the high-grade tFL samples, several genes associated with cellular cytotoxicity were also upregulated (*CXCL9/10/11, FCGR3A/B, GZMA/B/H*). Moreover, both the tumor suppressor *TP53* and its oncogenic P53-degradating counterpart *MDM2* were upregulated in tFL samples, as well as several apoptosis-related genes (*BCL2A1, BCL2L1* [BCL-xL], *CASP3*).

Based on the significant DEGs, PCA and hierarchal clustering could somewhat discriminate although not completely distinguish between the diagnostic st-FL samples and the high-grade tFL samples, as shown in Figure 3B,C. Notably, from the PCA, tFL samples showed more widespread presentations, indicating an increase in tumoral heterogeneity across the transformed samples. At the *p* < 0.01 significance threshold, 222 genes were identified. PCA based on this set of genes yielded almost identical clustering as the initial analyses, as shown in Supplementary Figure S1C. Likewise, a very similar performance was observed in the hierarchal clustering analyses, as shown in Supplementary Figure S1D.

### 3.5. Gene Enrichment Shows Possibly Disturbed Pathways from Diagnosis to Transformation

**Figure 4:**
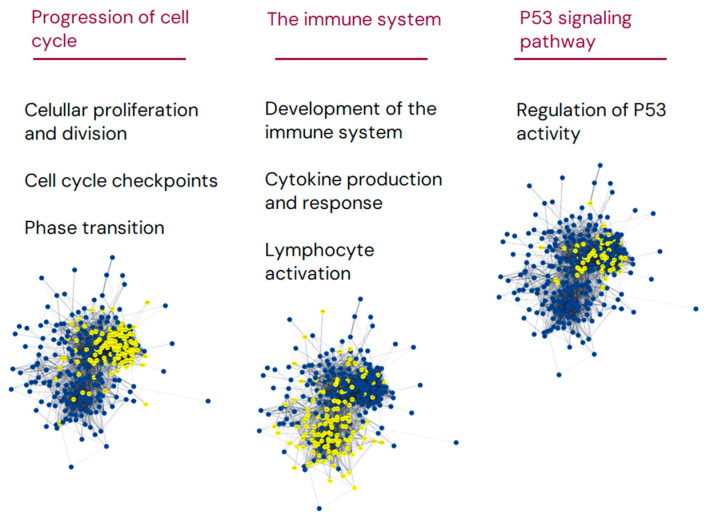
Gene enrichment analysis of significantly differentially expressed genes comparing st-FL and tFL samples. Gene enrichment analysis of the set of 314 DEGs comparing st-FL and tFL samples. Nodes represent proteins and edges visualize interactions. The depicted groups belong to the progression of the cell cycle, processes in the immune system, and P53 signaling. Yellow indicates proteins involved in the said pathway; blue are proteins not involved in the pathway.

The gene enrichment analysis was performed based on the set of 314 significant DEGs. From these, the STRING database recognized 313 nodes and 5368 edges, translating to a network with significantly more interactions than expected to occur at random (*p* < 0.001), as shown in Figure 4 and Table S4.

Based on the differential expression of this set of genes, the analyses highlighted changes in cellular pathways implicating different processes of cell cycle progression, the immune system, and the P53 signaling pathway. Altogether, these may indicate an influence on cellular growth and survival as well as changes in immune response processes, activation, differentiation, and regulation. Importantly, transformation from FL to tFL changes the tumoral cellular composition, and, therefore, such differences would be expected solely based on the type of cell analyzed.

### 3.6. In Silico Immunophenotyping Reveals Different Cellular Subgroups Associated with Outcome in FL

**Figure 5:**
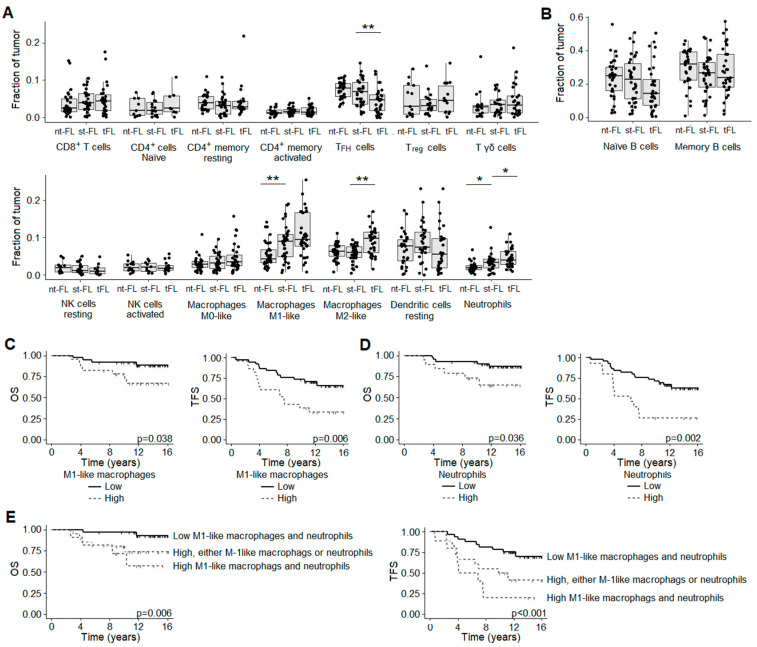
Cellular content of FL/tFL samples according to in silico immunophenotyping. (**A,B**) Immune cell infiltration in nt-FL, st-FL, and tFL lymphoma samples based on in silico immunophenotyping by CIBERSORTx and NanoString gene expression data. * *p* < 0.05, ** *p* < 0.01. (**C**) Outcome according to M1-like macrophage infiltrates in diagnostic nt-FL and st-FL samples. Left: analysis of overall survival (cutoff fraction 0.0709); right: analysis of transformation-free survival (cutoff fraction 0.0709). (**D**) Outcome according to neutrophil infiltrates in diagnostic nt-FL and st-FL samples. Left: analysis of overall survival (cutoff fraction 0.0267); right: analysis of transformation-free survival (cutoff fraction 0.0305). (**E**) Outcome analysis based on numbers of both M1-like macrophages and neutrophils, either one or both, dichotomized based on high and low cutoff from (**C**,**D**). Left: analysis of overall survival; right: analysis of transformation-free survival. Abbreviations: NK, natural killer; nt-FL, non-transforming FL; OS, overall survival; st-FL, subsequently transforming FL; TFH, T follicular helper; TFS, transformation-free survival; Treg, T regulatory.

To assess changes in the TME, we next performed in silico immunophenotyping using CIBERSORTx with its available signature matrix describing the expression fingerprints of 22 immune cell phenotypes [22]. Not surprisingly, the major cellular component of both FL (nt-FL and st-FL) and tFL samples were B cells, either the naïve or memory type, as shown in Figure 5A,B.

Several trends in the numbers of cellular subsets were seen in both the analyses between nt-FL versus st-FL samples, although they only reached statistical significance between the numbers of the proinflammatory M1-like type macrophages (*p* = 0.007) and neutrophils (*p* = 0.012), as shown in Figure 5A. Based on the number of M1-like macrophages, a significant difference in OS (*p* = 0.038) was observed for nt-FL and st-FL with high counts. This was even more evident in the analysis of TFS, which was significantly associated with high numbers of M1-like macrophages (*p* = 0.006), as shown in Figure 5C. The same was true in the analysis of neutrophil infiltrates in which high numbers were associated with inferior OS and TFS (*p* = 0.036 and *p* = 0.002, respectively), as shown in Figure 5D. Combining the counts of the two cellular subsets provided even better performance than when analyzed on their own, with inferior OS (*p* = 0.006) and TFS (*p* < 0.001) from high infiltrates of both M1-type macrophages and neutrophils in the lymphomas, as shown in Figure 5E. In the diagnostic st-FL and high-grade tFL samples, significant differences were seen with diminishing numbers of TFH cells (*p* = 0.007), as well as increasing numbers of immunosuppressive M2-like macrophages (*p* < 0.001) and neutrophils (*p* = 0.028), as shown in Figure 5A. Especially, this finding of reduced TFH and increased M2-like macrophage numbers in tFL was in accordance with the observed differences in gene expression as previously described. 

**Figure 6:**
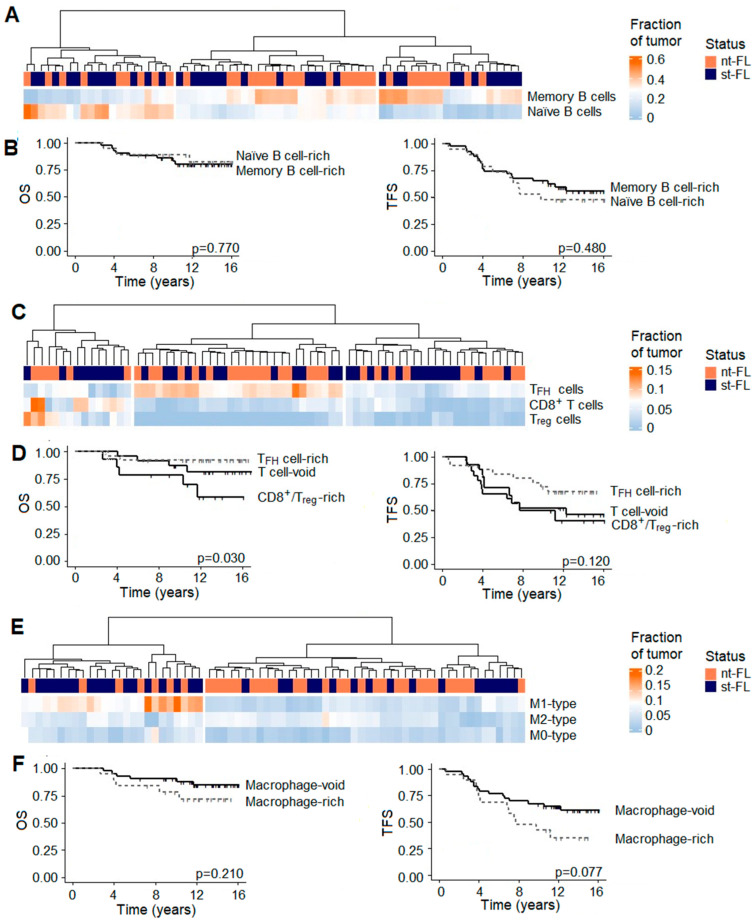
Cellular subgroups in diagnostic FL samples according to in silico immunophenotyping. Immune cell subsets in diagnostic lymphoma samples based on in silico immunophenotyping by CIBERSORTx and NanoString gene expression data. (**A**) Hierarchal clustering analysis based on B cell subtype proportions in diagnostic nt-FL and st-FL samples. (**B**) Kaplan–Meier survival analysis based on the dominating B cell subgroups. Left: analysis of overall survival; right: analysis of transformation-free survival. (**C**) Hierarchal clustering analysis based on T cell subtypes in diagnostic nt-FL and st-FL samples. (**D**) Kaplan–Meier survival analysis based on the dominating T cell subgroups. Left: analysis of overall survival; right: analysis of transformation-free survival. (**E**) Hierarchal clustering analysis based on macrophage subtypes in diagnostic nt-FL and st-FL samples. (**F**) Kaplan–Meier survival analysis based on the dominating macrophage subgroups. Left: analysis of overall survival; right: analysis of transformation-free survival. Abbreviations: NK, natural killer; nt-FL, non-transforming FL; OS, overall survival; st-FL, subsequently transforming FL; TFH, T follicular helper; TFS, transformation-free survival; Treg, T regulatory.

The in silico immunophenotyping hierarchical clustering separated nt-FL and st-FL samples into clusters based on their B cell subsets. The first cluster included patients with higher content of naïve B cells in the lymphoma tissues, while the others encompassed samples with higher contents of memory B cells; however, there were no difference in outcome between naïve-rich or memory-rich groups, as shown in Figure 6A,B. Additionally, clustering separated samples according to the T cell composition, with one group dominated by TFH cells, one group by CD8^+^ and Treg cells, and a group merely void of T cells, as shown in Figure 6C. The TFH-rich subset was predominated by nt-FL samples. Furthermore, OS and TFS were favorable in the TFH-rich group compared with the CD8^+^/Treg-rich group (*p* = 0.030 and *p* = 0.120, respectively), and they did not differ between the TFH-rich and T cell-void groups, as shown in Figure 6D. Lastly, based on the macrophage content in the lymphomas, two groups were identified. One group, primarily encompassing st-FL samples, had higher proportions of macrophages, while the other was almost void of macrophage subsets, as shown in Figure 6E. Based on these, the group with higher macrophage infiltration was associated with just trending inferior TFS (*p* = 0.077), as shown in Figure 6F.

## 4. Discussion

By gene expression profiling using a panel covering a broad range of genes involved in immunology and malignant disease, we characterized DEGs in diagnostic FL and high-grade transformed FL samples and their association with transformation. This study was performed as an explorative investigation with the aim of identifying novel markers holding predive value on the transformation of FL and elucidating currently unanswered aspects of tumor biology. 

We found that the expression of genes involved in B cell function, immunosuppressive, exhaustion, immune checkpoint processes, and macrophages were enriched in diagnostic samples from patients with subsequent transformation, while genes of T helper function were downregulated. Moreover, genes affecting important cellular pathways such as cell cycle, cellular migration, P53 function, and apoptosis were also differentially expressed, which was also highlighted in the gene enrichment analyses. However, as the GEP panel was restricted to including only pan-cancer and immune genes, perhaps it was not surprising that it is pathways such as these that we identified. By digital cytometry, we further demonstrated that increased counts of tumor-infiltration neutrophils and, quite surprisingly, M1-like macrophages in diagnostic samples had a detrimental prognostic role. This impact was particularly evident when the numbers of the two cellular subtypes were combined. At the time of transformation, high-grade tFL samples showed a decreased expression of T cell-related genes, while genes related to immune checkpoint molecules, exhaustion markers, and immunosuppressive M2-like macrophages were upregulated. This was further supported as immunophenotyping showed diminishing numbers of TFH cells as well as increasing M2-like macrophage counts. In addition, neutrophil count was further increased at the time of transformation. While the transformation process remains unclarified, together, these data emphasize that the intricate regulation of the TME plays an important role in the deregulation of genes important for helper T cell function and immunosuppression. The immune microenvironment is believed to influence the clinical behavior in the disease, such as high T cells that are known to correlate with improved rituximab treatment, and, correspondingly, immune infiltrates collectively constitute a large proportion of the total tumor composition. In our study, we found that B cells compose the largest cell subset compartment. While thoroughly validated, possible misinterpretations from the estimated cell fractions must be considered, as immunophenotyping was performed in silico. This is especially relevant, as disagreements between the genotype and functional cellular phenotype might occur depending on multiple factors. 

While not yet abundantly studied in regard to FL, increased awareness of tumor-associated macrophages has been raised, as they may serve as mediators in important functions of malignant transformation, progression, angiogenesis, and modulation of antitumor immunity [28]. In addition to increased neutrophil numbers, genes important for neutrophils were found upregulated in st-FL samples, including *CD47, CEACAM1* [CD66], *CSF-R3, ICAM1*, and *IL6*, the latter a cytokine which is also important for the maturation of B cells. In addition, these molecules play essential roles in neutrophilic maturation, including proliferation, differentiation, and survival. Moreover, they function in adhesion and recognition events at the cell surface [28,29,30]. Interestingly, expression levels of activation markers, such as CD11a and CD11b, are increased in the neutrophils, of which the latter is known to interact with the CD44 of malignant B cells and thus inhibit apoptosis [31]. The results from the present study, in particular, show that *CD44* expression was increased in the st-FL tumor along with several members of the CD11a/b family (*ITGA1, ITGA5, ITGB1, ITGAV*), and all integrins were involved in cellular adhesion and migration [28,32]. We previously explored this signaling axis at the protein level by investigation of CD44 and Receptor for Hyaluronan Mediated Motility (RHAMM) in FL biopsies, in which the latter were predictive of transformation [33]. Supportive of this, genes encoding important proteins in this signaling axis were also identified in the present study, including *AURKA, IL6*, and *SERPINE1* [PAI-1] [34,35,36]. The previous study was performed using samples from patients diagnosed before rituximab was introduced as standard treatment [33]. Thus, further investigation into the CD44/RHAMM signaling axis would be worth performing in relation to the transformation of FL. In addition, we also previously identified the CD11a protein in a large-scale proteomics study, in which, among others, cytoskeletal rearrangement, cellular adhesion, and migration were found important in the transformation process [27]. Interconnected by changes to the cytoskeleton, mounting evidence implicates the cytoskeleton to play a key role as a mediator and regulator of apoptosis as well as the progression of the cell cycle [37]. Other gene-encoding drivers of the cell cycle, including *ATR, CCNB2/E1/F/K, CDC25A, CDK4, CDK6, CDKN1A, CHEK1,* and *UBE2C*, were also identified in the present study, of which the CDK6 protein was also identified at the protein levels in the previous proteomics study [27]. 

Emerging concepts of neutrophil heterogeneity and plasticity have revealed that neutrophils may differentiate into discrete subsets defined by distinct function characteristics, which adds to the already complex interaction between neutrophils and other immune cell types [28,30]. One of these interacts with macrophages, which, under physiological conditions, enables the host to efficiently defend against foreign pathogens. However, it has also become increasingly evident that this interaction can be detrimental if not tightly regulated. In fact, this cooperation of neutrophils and macrophages may regulate the direction of the immunological response by polarizing M1-like or M2-like macrophages, respectively [29,30,32,38]. While molecules such as CD14 and CD68 are reported as pan-macrophage markers, rather surprisingly, digital cytometry found M1-like macrophages, commonly known as proinflammatory or antitumoral macrophages, to be upregulated in subsequently transforming samples. In the present study, we found the upregulation of the *CCL2* gene, encoding a chemokine ligand that exerts chemotactic activity while favoring the recruitment of macrophages to the tumor [31]. Tumor-associated macrophages are often polarized to the M2-like type in response to IL-4, IL-10, IL-13, and other cytokines, favoring angiogenesis, dissemination, and immunosuppression [31,39]. They cooperate with the T cell-derived CD40 ligand to promote B cell proliferation, of which the encoding *CD40LG* gene conversely was found downregulated in st-FL samples [31]. Collectively, this could indicate an increased host response against the tumor with infiltrates of proinflammatory macrophages and a decrease in T helper function. At the time of transformation, the expression of the *CD163* gene, specific to the immunosuppressive M2-like type, was increased in tFL samples, as well as a further downregulation of genes involved in T helper function. Thus, as the st-FL lymphomas eventually transform, other deregulated processes will have to take place to evade such antitumoral responses, as indicated by the changes observed in the paired high-grade samples.

Our data extend previous findings of gene expression in FL, which are demonstrated in patients all diagnosed in the rituximab era. The TME houses several types of immune cells, and the contribution of these to patient outcomes was first described by Dave et al. [12]. However, studies have later shown conflicting results, sometimes depending on incorporated treatment regimens, with some not able to reproduce the prognostic value [40,41,42]. Gene expression profiling has been conducted before at different settings and aims in FL [12,14,16,40,41,42,43,44,45,46,47,48,49]. A caveat, however, for the interpretation of these results, is that the majority of studies primarily included patients diagnosed before the introduction of rituximab treatment. Rituximab treatment has improved FL prognosis and risk of transformation. In relation to this, previous studies have primarily focused on outcome (i.e., overall survival, event-free survival, etc.), whereas we sought to gain insight into the risk of subsequent transformation. Nevertheless, in investigating FL transformation, Gentles et al. [16] used gene expression data linking a pluripotency/embryonic stem cell-like signature to an increased risk of transformation [16,50]. From these data, the authors suggested that transformed FL might arise from a more differentiated germinal center FL progenitor clone that acquires an enhanced stem cell-like expression pattern [16]. Moreover, Brodtkorb et al. [13] described a gene expression signature of NFκB-related genes predictive of subsequent transformation, which was later validated by Steen et al. [13,17]. B cell receptor activation and NFκB signaling are indeed important in the maturation and function of B cells. Accordingly, in the present study, we also identified DEGs at diagnosis between nt-FL and st-FL samples related to B cell function, activation, maturation, and BCR-NFκB signaling, including *CD40, HLA-DRB1, IL6, IRF4* [MUM1], and *RELB* [NFκB].

Prognostic effects of single genes have also been investigated, primarily investigating survival outcomes and less so transformation. Tobin et al. [51] reported differences in the expression of immune checkpoints. Particularly, low levels of immune markers would identify patients enriched for early progression, with *PDCD1LG2* [PD-L2] as the marker with the highest accuracy [51]. The inverse of that was found in the present study with a borderline significant increase in *PDCD1LG2* [PD-L2] between st-FL and nt-FL samples, as well as an even further increase in tFL samples. Moreover, we found borderline significant increases in *PDCD1* [PD-1] expression in st-FL samples compared with nt-FL samples, followed by significantly decreased levels of the gene in high-grade tFL samples. We previously investigated the *PDCD1*-encoded programmed death 1 (PD-1) protein, which showed the same tendencies [52]. Yet, over time, especially analyses of PD-1 have yielded quite conflicting results, and thus, the possible effects of this marker—pre- or post-rituximab—remain not fully elucidated [52,53,54,55,56]. 

The mechanisms driving lymphoma transformation are undoubtedly complex and heterogeneous, as also reflected by the presented gene expression data. Data on gene expression poses a challenge to interpretation due to the combinatorial nature of transcriptional regulation, which must be considered highly interconnected. Moreover, the present study was performed using bulk tissue material without distinguishing between different cellular structures. Such studies must be regarded as hypothesis generating, and the results from such studies must be further evaluated by other more in-depth analyses in the future. Importantly, the presently studied cohort included more men to women in the st-FL group compared with the nt-FL group. Thus, it cannot be ruled out that some of the differences found could be based on gender, and thus, these will need further investigation. Another essential aspect is the fact that the tFL biopsies included in the present study were obtained after treatment (radiation, rituximab, cytotoxic treatment, etc.), and thus, the observed differences in these biopsies could reflect a tumoral response to treatment.

## 5. Conclusions

In a disease where the non-malignant microenvironment plays such a fundamental role, better characterization of the immune profiles in different clinical outcomes remains warranted. Our results provide insights into the interplay between neoplastic and bystander cells of the TMA, particularly demonstrating a novel adverse impact of neutrophils and macrophages on transformation in FL. Explorative in nature, the presented results may aid in the discovery of putative predictive markers and identify new candidate targets aiding in tailoring personalized immunotherapies accordingly.

## Figures and Tables

**Table 1:** Patients’ clinicopathological features.

Characteristics	All n = 70 n (%)	nt-FL n = 34 n (%)	st-FL n = 36 n (%)	*p*-Value
Sex Male Female	37 (53) 33 (47)	13 (38) 21 (62)	24 (67) 12 (33)	0.032
Age at FL diagnosis Median Range	58 35–80	54 35–74	59 39–80	NS
Ann Arbor stage I–II III–IV	24 (34) 46 (66)	11 (32) 23 (68)	13 (36) 23 (64)	NS
FLIPI Low Intermediate High	28 (40) 17 (24) 25 (36)	15 (44) 8 (24) 11 (32)	13 (36) 9 (25) 14 (39)	NS
LDH elevation Yes No Unknown	16 (23) 51 (73) 3 (4)	5 (15) 28 (82) 1 (3)	11 (31) 23 (64) 2 (6)	NS
B symptoms Yes No	15 (21) 55 (79)	9 (26) 25 (74)	6 (17) 30 (83)	NS
Performance score <2 ≥2 Unknown	69 (99) 0 (0) 1 (1)	34 (100) 0 (0) 0 (0)	35 (97) 0 (0) 1 (3)	NS
Bone marrow involvement Yes No Unknown	22 (31) 47 (67) 1 (1)	12 (35) 22 (65) 0 (0)	10 (28) 25 (69) 1 (3)	NS
Anemia Yes No Unknown	8 (11) 61 (87) 1 (1)	3 (9) 30 (88) 1 (3)	5 (14) 31 (86) 2 (6)	NS
FL histology FL NOS FL grade 1–2 FL grade 3A	7 (10) 55 (79) 8 (11)	5 (15) 26 (76) 3 (9)	2 (6) 29 (81) 5 (14)	NS
Number of nodal sites ≥5 <4	30 (43) 40 (57)	17 (50) 17 (50)	13 (36) 23 (64)	NS
Extranodal disease Yes No	26 (37) 44 (63)	12 (35) 22 (65)	14 (39) 22 (61)	NS
Bulky disease * Yes No Unknown	15 (21) 52 (74) 3 (4)	8 (24) 25 (74) 1 (3)	7 (19) 27 (75) 2 (6)	NS

* Bulky disease was defined as a maximum tumor diameter of >6 cm.

## Data Availability

Data will be available upon reasonable request to the corresponding author.

## Supplementary Materials

The following supporting information can be downloaded at https://www.mdpi.com/article/10.3390/cancers16203553/s1, Figure S1: Clustering analyses in diagnostic and transformed samples based on a lower significance threshold; Table S1: 647 analyzed genes comparing st-FL and nt-FL samples; Table S2: Complete STRING pathway analysis comparing nt-FL and st-FL samples; Table S3: 659 analyzed genes comparing tFL and st-FL samples; Table S4: Complete STRING pathway analysis comparing st-FL and tFL samples.
