# Supplementary material for: Impact of the Immune Landscape in Follicular Lymphoma: Insights into Histological Transformation in the Rituximab Era
Source: Cancers (Basel). 2024 Oct 21;16(20):3553. doi: 10.3390/cancers16203553 (PMC11506075; doi:10.3390/cancers16203553)
Supplement: Supplementary file 1 [file cancers-16-03553-s001.zip › Supplementary material.pdf]

Supplementary tables

Table S1: 647 analyzed genes comparing st-FL and nt-FL samples

Table S2: Complete STRING pathway analysis comparing nt-FL and st-FL samples

Table S3: 659 analyzed genes comparing tFL and st-FL samples

Table S4: Complete STRING pathway analysis comparing st-FL and tFL samples

Supplementary Figures

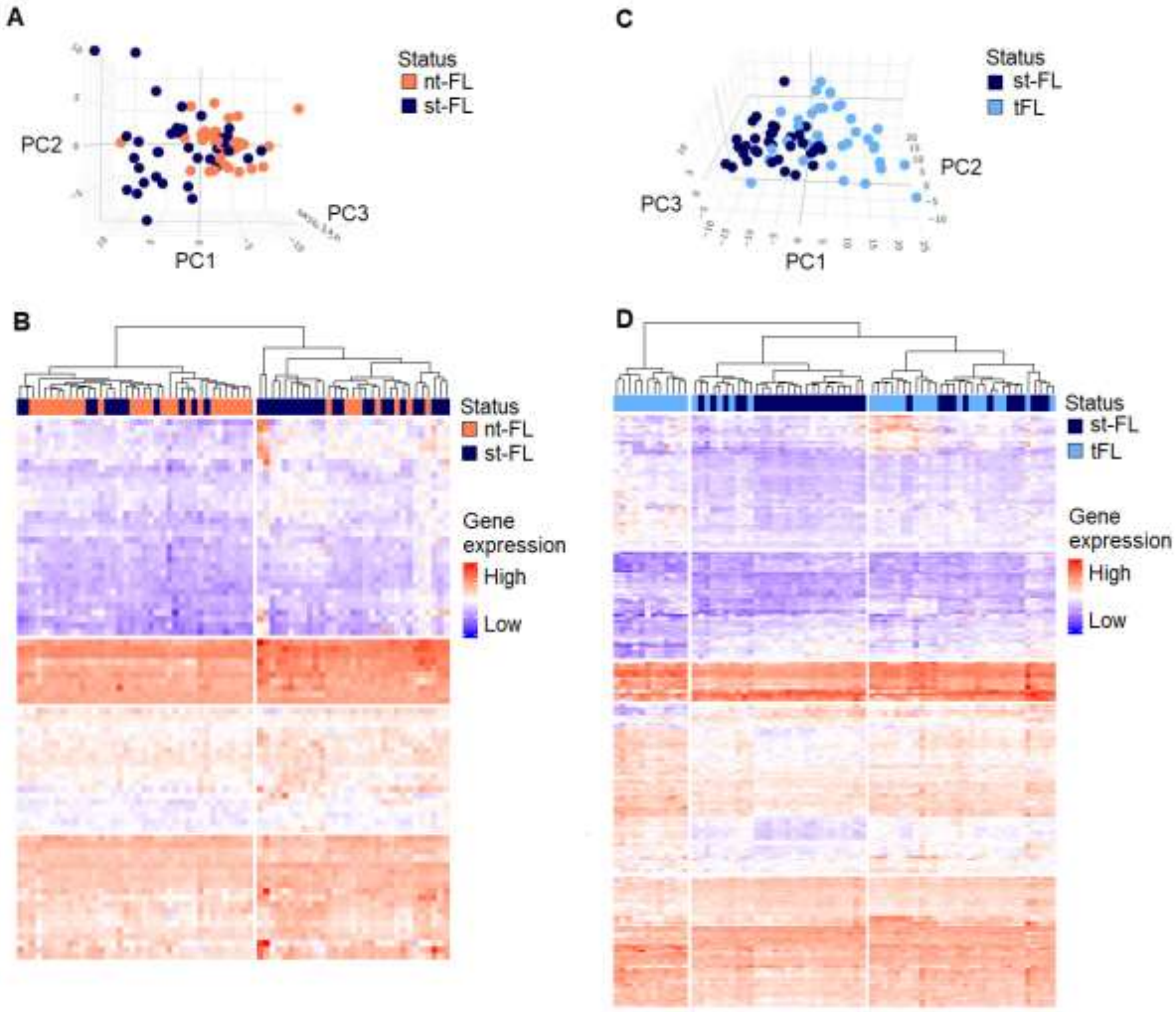

Supplementary Figure S1: Clustering analyses in diagnostic and transformed samples based on a lower significance threshold

(A-B) 3D PCA plot and hierarchal clustering analysis with input of differentially expressed proteins at p<0.01 comparing nt-FL and st-FL samples. (C-D) 3D PCA plot and hierarchal

clustering analysis with input of differentially expressed proteins at $p<0.01$ comparing st-FL and tFL samples.

Abbreviations: nt-FL, non-transforming samples; PC, principal component; st-FL, subsequently-transforming samples; tFL, transformed FL.
